# Longitudinal associations between socioeconomic status and cardiovascular disease in a Chinese population: Evidence from CHARLS

**DOI:** 10.1371/journal.pone.0328924

**Published:** 2025-08-22

**Authors:** Qingping Zeng, Mengqian Liao, Yu Li, Fei She, Ping Zhang

**Affiliations:** 1 Department of Cardiology, Beijing Tsinghua Changgung Hospital, School of Clinical Medicine, Tsinghua Medicine, Tsinghua University, Beijing, China; 2 Heart Center, The First Hospital of Tsinghua University (Beijing Huaxin Hospital), School of Clinical Medicine, Tsinghua University, Beijing, China; 3 Dalian Medical University, Dalian, China; The First Affiliated Hospital of Soochow University, CHINA

## Abstract

**Importance:**

The relationship between socioeconomic status and cardiovascular disease among the general Chinese population is inconclusive.

**Objective:**

This study aims to investigate the relationship between socioeconomic status and cardiovascular disease in the Chinese general population through a large sample.

**Design:**

We retrospectively analyzed data from the 2015 and 2018 waves of the China Health and Retirement Longitudinal Study (CHARLS). Participants were required to be at least 45 years old and have cardiovascular disease (CVD) data. The study was divided into two parts: a cross-sectional analysis using 2015 data, and a longitudinal analysis of participants without CVD in 2015 but with complete follow-up data in 2018. Socioeconomic status (SES) was assessed using self-reported household income, occupation, education level, and health insurance. CVD events were identified through participant-reported diagnoses of heart disease or stroke. Logistic and Cox proportional hazards regression models were used to estimate the association between SES and CVD.

**Participants:**

16,560 participants were included in the cross-sectional analysis and 11,587 in the longitudinal analysis.

**Exposures:**

Socioeconomic status.

**Main outcomes and measures:**

Cardiovascular disease, heart disease, and stroke.

**Results:**

In the cross-sectional analysis, 16% of the 16,560 participants had CVD, with higher prevalence in lower SES groups. After adjustments, middle SES was significantly associated with increased CVD risk (OR 1.77, 95% CI 1.21–2.58). In the longitudinal analysis, during a 3-year follow-up, 12.1% of 11,587 participants developed CVD. Middle SES was associated with a 67% higher risk of new-onset CVD (HR 1.67, 95% CI 1.02–2.74). Sensitivity analysis confirmed these findings, with middle SES showing a significant association with CVD risk (HR 1.32, 95% CI 1.04–1.67).

**Conclusions and relevance:**

In the general population of China, middle socioeconomic status is positively associated with cardiovascular disease and is more likely to be associated with new-onset cardiovascular disease. Our findings support the need for trade-offs between socioeconomic status groups to benefit different populations, especially considering the middle socioeconomic status group, which is an easily overlooked group. However, more long-term prospective studies are needed to further elucidate the relationship between changes in socioeconomic status and cardiovascular disease in China.

## Introduction

Global deaths from cardiovascular disease (CVD) continue to climb, and cardiovascular disease is a major contributor to the disproportionate costs of global health systems [1–4]. The promotion of cardiovascular health not only helps individuals to avoid potential health crises, but also helps to reduce the economic burden on families and societies and to reduce mortality from non-communicable diseases worldwide.

There are significant regional and national differences in cardiovascular disease, with more than 75% of the global CVD burden concentrated in low- and middle-income countries [5]. Considering that different populations may face different risk factors, have different health needs and access to resources. As well, different populations may have different knowledge and awareness of CVD prevention [6–9]. Implementing customized prevention strategies for different segments of the population can more effectively address the diverse risk factors for CVD and improve the coverage and effectiveness of preventive measures, thereby reducing the global burden of CVD.

Socioeconomic status (SES) is a multidimensional concept that includes economic, social, and social class factors. These factors interact with each other and together define a person’s or family’s place in the social structure [10]. A number of studies have reported the presence of SES inequalities in CVD patients, and the role played by SES in cardiovascular health is gradually gaining interest [11,12]. Evidence from family-based studies and Mendelian randomization studies supports a causal effect of low SES on cardiovascular disease in high-income countries [13]. However, more high-quality research evidence is still needed to verify the reliability of this idea.

The association between SES and CVD may be influenced by a variety of factors, including psychological, genetic, environmental, etc. [14–16]. SES may affect CVD risk in different ways at different life stages. As the result of differences in economic status, per capita health care expenditures, and prevention policies in each country, an in-depth understanding of the role of SES on CVD in different countries may lead to more effective identification of populations at high cardiovascular risk and more targeted measures to reduce potential health disparities. Further research in countries with different income levels is therefore necessary.

Low SES was associated with higher CVD risk in 2 large cohort studies based on US and UK populations [17]. In the Polish study, SES was recognized as an independent predictor of high CVD mortality risk. In the Greek population-based study, SES was shown to be a strong predictor of CVD incidence [18]. These findings underscore the need to incorporate SES into CVD risk calculations and screening, and to reduce the impact of behavioral and psychosocial risk factors, especially for populations associated with lower socioeconomic status to detect cardiovascular disease early.

China has experienced significant socioeconomic and demographic changes driven by industrialization and urbanization [19]. The growing burden of cardiovascular disease (CVD) is a global public health concern. In China, rapid urbanization, income disparities, and unequal healthcare access have created unique socioeconomic contexts that shape CVD risk factors and outcomes. Despite significant advancements in cardiovascular care, these disparities persist, particularly between rural and urban populations. The need for targeted interventions is urgent, especially as China grapples with a rapidly aging population and an increasing prevalence of CVD [20,21]. To better understand these disparities, global studies such as the Prospective Urban Rural Epidemiology (PURE) study have highlighted the critical role of socioeconomic status (SES) in CVD outcomes [22]. These studies provide valuable insights into the complex relationship between SES and CVD, which can inform policies aimed at addressing health inequities in China. While China, one of the largest developing countries, is undergoing rapid economic growth, urbanization, and demographic aging, all of which have contributed to an increased risk of cardiovascular diseases [23]. And This study aims to further explore the association between SES and CVD in a large sample of general Chinese population, which will help us to further understand the relationship between SES and CVD.

## Methods

The China Health and Retirement Longitudinal Study (CHARLS) is a national survey of Chinese residents aged 45 and older, launched in 2008. It aims to comprehensively understand aging in China, including demographics, health, socioeconomic status, and retirement [24,25]. The 2011 baseline survey included over 17,000 individuals from 10,000 households across 28 provinces, using multistage probability sampling with biennial data collection. CHARLS data are publicly available for research, providing insights into health and economic impacts of rapid aging in China. For more details, see Zhao et al. (2014) in the International Journal of Epidemiology.

**Fig 1:**
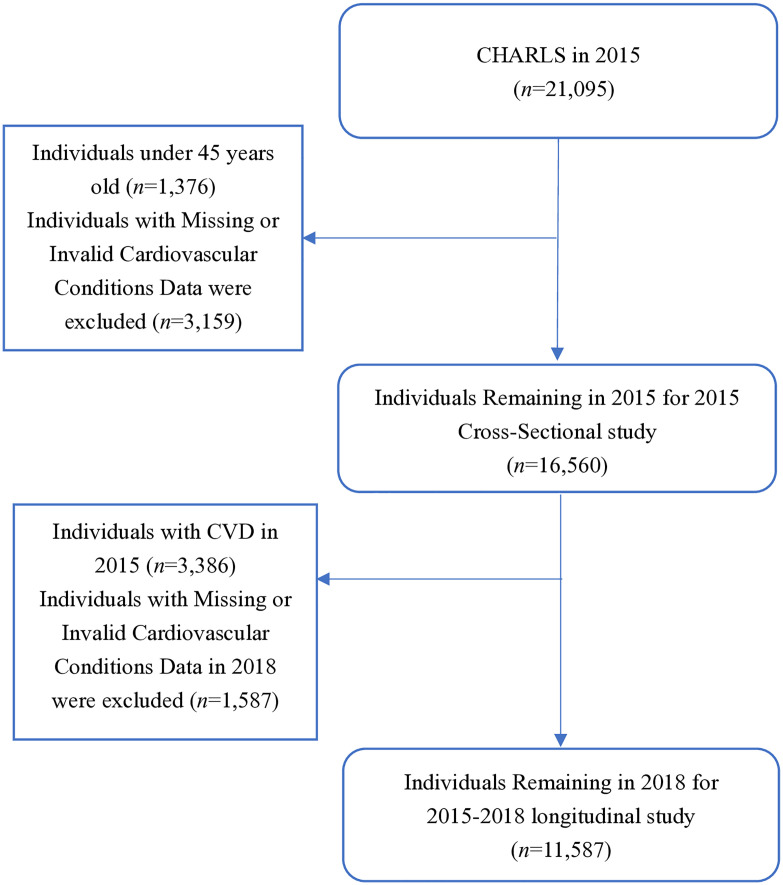
Participant flowchart.

Data from the 2015 and 2018 CHARLS surveys were retrospectively analyzed [26]. Inclusion criteria were: 1) individuals aged at least 45 in CHARLS 2015; 2) availability of cardiovascular disease (CVD) data (including heart disease and stroke). Exclusion criteria were: 1) missing CVD data in CHARLS 2015; 2) missing age information; 3) individuals under 45 years old. The study is divided into two parts:1. Cross-sectional analysis: Using data from the 2015 follow-up cohort. In CHARLS 2015, 21,095 participants were interviewed. We excluded 4,535 participants due to missing CVD data (heart disease n = 4535, stroke n = 116), missing age information (n = 47), and being under 45 years old (n = 240), leaving 16,560 participants for cross-sectional analysis. 2. Longitudinal analysis: We further excluded 3,386 participants with CVD in 2015 and 1,587 participants without CVD data in 2018. The final sample included 11,587 participants who were free of CVD in 2015 and had complete follow-up data in 2018. The detailed selection process is shown in Fig 1.

Written informed consent was signed by all participants or their legal guardians for both the initial and subsequent surveys. Consent was secured prior to filling out the study questionnaire. The Peking University Biomedical Ethics Review Committee (IRB00001052–11015) approved this study [27].

### SES assessment

CHARLS measured SES based on self-reported household income, occupation, education, and health insurance. Based on practical interpretation and sample size within levels, factors were categorized into three levels (low, middle, high). Education levels were divided into primary or below, secondary, and college or above. Household income was divided into tertiles of post-tax earnings: high (third tertile), middle (second tertile), and low (first tertile). Occupations were categorized as non-agricultural workers, agricultural workers, and unemployed (including unemployed, retired, and never worked). Health insurance is categorized into private health insurance, public health insurance only, and no health insurance. A comprehensive SES variable was created using latent class analysis (LCA), combining household income, occupation, education level, and health insurance (each divided into three levels). We categorized SES based on four dimensions: education, insurance, income, and occupation. Each dimension was divided into three levels (high, middle, and low). Subsequently, we used Latent Class Analysis (LCA) to generate the final SES groups [17]. This analysis used the poLCA package in R, identifying three latent classes representing high, middle, and low SES [28].

### CVD events assessment

CVD events, including heart disease and stroke, were evaluated using questions from previous studies. Participants were asked if a doctor had ever diagnosed them with conditions like heart attack, angina, coronary heart disease, heart failure, or other heart issues, and whether they had ever been diagnosed with a stroke. Those who reported either heart disease or stroke were classified as having CVD [29–31].

### Covariates

Sociodemographic variables included age, gender, marital status (married or other), and residence (rural or urban) [27,32]. Health-related factors included body mass index (BMI), smoking and drinking status (yes or no), vigorous exercise for at least 10 minutes (yes or no), hypertension, diabetes, dyslipidemia, and self-reported physician-diagnosed kidney disease. Hypertension was defined as systolic blood pressure ≥130 mmHg and diastolic blood pressure ≥80 mmHg or current use of any antihypertensive treatment [33]. Diabetes was defined based on a physician’s diagnosis, use of diabetes medication, or plasma glucose ≥200 mg/dL [34]. Dyslipidemia was defined based on self-reported physician diagnosis and use of lipid-lowering medication. BMI was categorized into three groups: underweight (BMI < 18.5 kg/m²), normal weight (BMI 18.5–24.9 kg/m²), and overweight or obese (BMI ≥ 25 kg/m²) [35].

In the cross-sectional analysis, a subset of 10,917 individuals had metabolic biomarkers measured, including total cholesterol, triglycerides, LDL cholesterol, HDL cholesterol, and serum creatinine.

### Statistical analysis

Data for continuous variables are presented as mean ± standard deviation (SD), while categorical variables are shown as sample size and percentage for each value. Baseline characteristics in the cross-sectional and longitudinal analysis samples were summarized by SES groups. Statistical comparisons were performed using appropriate methods: categorical variables were analyzed using chi-square tests, and continuous variables were analyzed using ANOVA. In the cross-sectional analysis, Logistic regression analysis was employed to estimate the association between SES and CVD and its components. In the longitudinal analysis, the incidence of CVD per 1,000 person-years in CHARLS 2018 was calculated. Follow-up time was measured from the last interview date to the date of CVD diagnosis or the most recent interview (March 2019). Cox proportional hazards models was employed to estimate the relationship between baseline SES and new CVD events, hazard ratios (HRs) and 95% confidence intervals (CIs) were calculated. Sensitivity analyses were conducted by reproducing all regressions based on marginal structural models (MSM).

In both cross-sectional and longitudinal analyses, three models were estimated: Model 1 adjusted for age, gender, marital status, and residence; Model 2 adjusted for age, gender, marital status, residence, smoking, drinking, and exercise; Model 3 adjusted for Model 2 variables plus dyslipidemia, BMI, hypertension, diabetes, and chronic kidney disease. Additionally, metabolic biomarkers were further adjusted for in a subset of participants who underwent metabolic testing (10,917 participants for cross-sectional and 7,910 for longitudinal analysis). All statistical analyses were performed using R version 4.3.2 (R Foundation for Statistical Computing) and Empower 6.0. A P-value < 0.05 was considered statistically significant in all cases.

## Result

### Baseline characteristics

Table 1 presents the characteristics of all 16,560 participants categorized by SES. The average age (SD) of the study population was 61.3 (9.8) years, with 52.0% being female (8619/16,560). Among these 16,560 middle-aged and older adults, 7.1% (1172) were classified as high SES, 60.1% (9949) as middle SES, and 32.8% (5439) as low SES. Compared to the high and low SES groups, individuals in the middle SES group tended to be older, had more women, less private insurance, more people with less than a high school education, fewer non-agricultural workers, higher income, more urban residents, less smoking and drinking, less physical activity, higher total cholesterol, and more cases of hypertension and CVD (including heart disease and stroke) (all P < 0.01).

**Table 1:** Baseline characteristics of all participants by socioeconomic status.

Characteristics	Total (n = 16560)	socioeconomic status			P-value
		High (n = 1172)	Middle (n = 9949)	Low (n = 5439)	
Age(y)	61.3 ± 9.8	58.5 ± 8.9	63.8 ± 10.0	57.3 ± 8.0	<0.001
Gender					<0.001
Man	7941(48.0%)	573(48.9%)	4024(40.4%)	3344(61.5%)	
Woman	8619(52.0%)	599(51.1%)	5925(59.6%)	2095(38.5%)	
Health insurance					<0.001
No	1435(9.0%)	784(69.1%)	625(6.4%)	26(0.5%)	
Public	14207(88.8%)	0(0.0%)	9104 (93.5%)	5103(99.4%)	
Private	360 (2.2%)	350(30.9%)	3 (0.0%)	7(0.1%)	
Occupation					<0.001
Unemployment or retirement	6055(36.9%)	169(14.5%)	5140(51.7%)	746(14.1%)	
Agriculture	5843(35.6%)	540(46.4%)	4711(47.4%)	592(11.2%)	
Nonagriculture	4513(27.5%)	456(39.1%)	89 (0.9%)	3968 (74.8%)	
Educational level					<0.001
Less than high school	14627(88.3%)	1003(85.6%)	9702(97.5%)	3922(72.1%)	
High school or equivalent	1607(9.7%)	142(12.1%)	0(0.0%)	1465(26.9%)	
College or above	326(2.0%)	27(2.3%)	247(2.5%)	52(1.0%)	
Incomes category					<0.001
Low	1238(39.6%)	67(21.5%)	25(12.8%)	1146(43.7%)	
Medium	1019(32.6%)	112(36.0%)	0(0.0%)	907(34.6%)	
High	873(27.9%)	132(42.4%)	170(87.2%)	571(21.8%)	
Marital status					<0.001
Married	13247(80.0%)	966 (82.4%)	7742(77.8%)	4539(83.5%)	
Partnered	952(5.7%)	60(5.1%)	446(4.5%)	446(8.2%)	
Separated	34(0.2%)	4(0.3%)	18(0.2%)	12 (0.2%)	
Divorced	129(0.8%)	17(1.5%)	64(0.6%)	48(0.9%)	
Widowed	2079(12.6%)	108(9.2%)	1601(16.1%)	370(6.8%)	
Never married	119(0.7%)	17(1.5%)	78(0.8%)	24(0.4%)	
Residence					<0.001
Urban community	6361(38.4%)	479(40.9%)	3377(33.9%)	2505(46.1%)	
Rural village	10199(61.6%)	693(59.1%)	6572(66.1%)	2934(53.9%)	
BMI category					<0.001
Underweight	809(6.2%)	52(5.6%)	594(7.3%)	163(4.0%)	
Normal weight	6374(48.6%)	477(51.3%)	3979 (48.7%)	1918(47.7%)	
Overweight or obesity	5937(45.3%)	400(43.1%)	3593(44.0%)	1944(48.3%)	
Smoking					<0.001
No	9185(55.5%)	652(55.7%)	5959 (59.9%)	2574(47.4%)	
Yes	7368(44.5%)	519(44.3%)	3990(40.1%)	2859(52.6%)	
Drinking					<0.001
No	8909 (53.9%)	568(48.5%)	5895(59.4%)	2446(45.1%)	
Yes	7619 (46.1%)	602(51.5%)	4037(40.6%)	2980(54.9%)	
Physical Activity					<0.001
No	5135(65.7%)	315(58.8%)	3229(69.1%)	1591(61.1%)	
Yes	2675(34.3%)	221(41.2%)	1443(30.9%)	1011(38.9%)	
Triglycerides (mg/dl)	142.7 ± 90.7	141.0 ± 93.7	141.4 ± 88.7	146.0 ± 94.0	0.048
Creatinine (mg/dl)	0.8 ± 0.3	0.8 ± 0.2	0.8 ± 0.3	0.8 ± 0.3	0.006
HDL cholesterol (mg/dl)	51.3 ± 11.7	52.2 ± 12.1	51.5 ± 11.7	50.7 ± 11.5	<0.001
LDL cholesterol (mg/dl)	102.7 ± 29.2	102.0 ± 28.5	103.5 ± 29.4	101.1 ± 28.7	<0.001
Total cholesterol (mg/dl)	184.5 ± 36.8	184.1 ± 34.6	185.6 ± 37.5	182.5 ± 35.6	<0.001
Glucose (mg/dl)	104.1 ± 36.2	102.3 ± 33.2	104.5 ± 36.4	103.8 ± 36.5	0.244
Hypertension					<0.001
No	5723(41.6%)	453(47.2%)	3375(39.4%)	1895(44.9%)	
Yes	8025(58.4%)	507(52.8%)	5193(60.6%)	2325(55.1%)	
Dyslipidemia					0.115
No	13033(81.4%)	940(83.2%)	7761(80.9%)	4332(81.8%)	
Yes	2985(18.6%)	190(16.8%)	1830(19.1%)	965(18.2%)	
Heart diseases					<0.001
No	13599(82.1%)	1011(86.3%)	7892(79.3%)	4696(86.3%)	
Yes	2961(17.9%)	161(13.7%)	2057(20.7%)	743(13.7%)	
Stroke					<0.001
No	15918(96.1%)	1134(96.8%)	9474(95.2%)	5310(97.6%)	
Yes	642(3.9%)	38(3.2%)	475(4.8%)	129(2.4%)	
CVD					<0.001
No	13174(79.6%)	984(84.0%)	7578(76.2%)	4612(84.8%)	
Yes	3386(20.4%)	188(16.0%)	2371(23.8%)	827(15.2%)	
Kidney Diseases	14822 (90.2%)	1058 (91.2%)	8858 (89.8%)	4906 (90.6%)	0.146
No	1612 (9.8%)	102 (8.8%)	1002 (10.2%)	508 (9.4%)	
Yes					

Data are shown as means ± standard deviation or numbers (percentages).

Abbreviation: BMI, body mass index; LDL, low-density lipoprotein; HDL, high-density lipoprotein; CVD, Cardiovascular disease. Categorical variables were analyzed using chi-square tests, and continuous variables were analyzed using ANOVA.

S1 Table shows the baseline characteristics of the 11,587 participants without cardiovascular disease in 2015 (51.3% female; average age 60.2 ± 9.3 years), stratified by SES.

### Cross-sectional association between ses and cardiovascular disease

**Fig 2:**
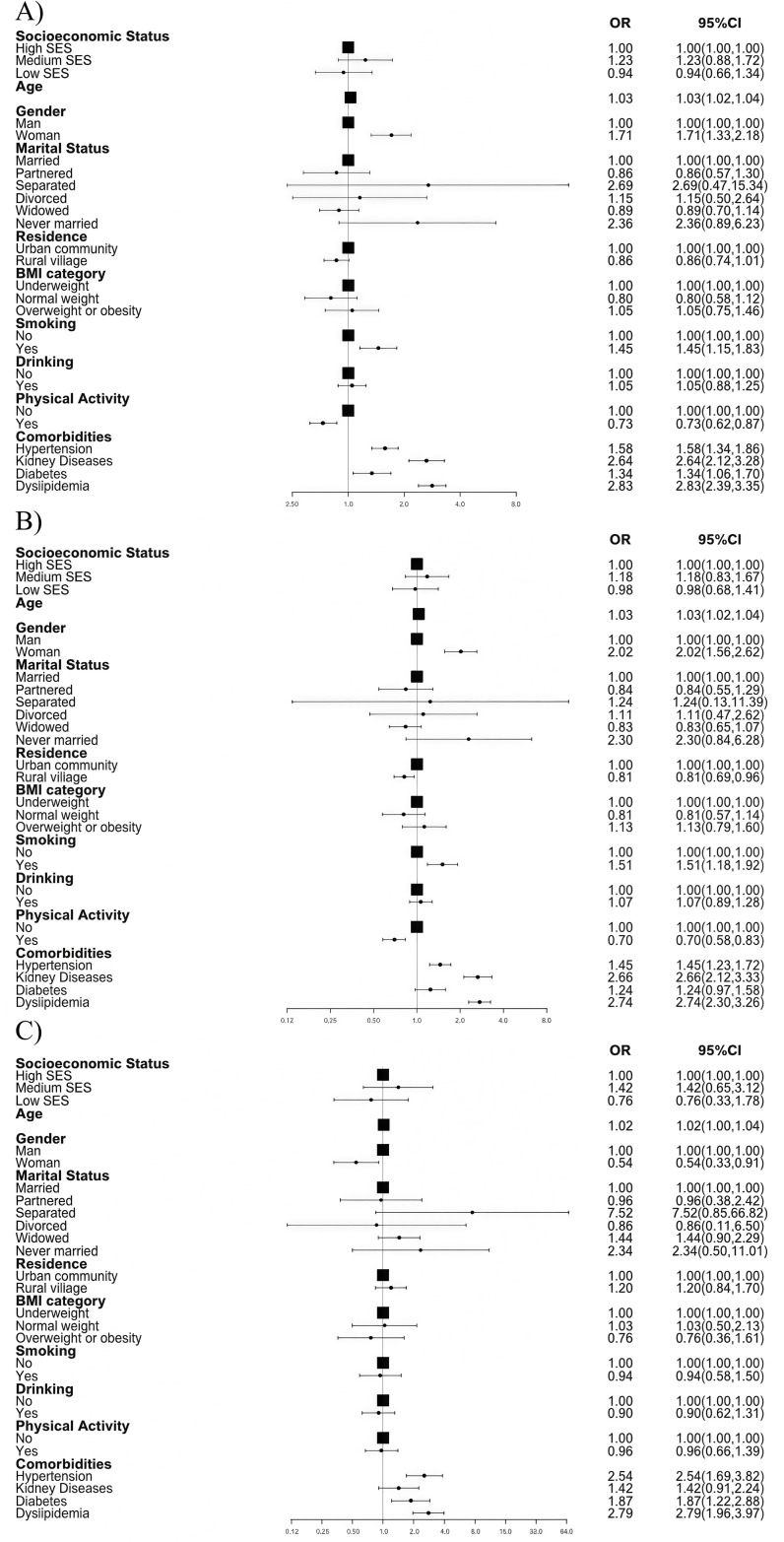
ORs and 95% CIs for CVD and its components by socioeconomic status in cross-sectional analyses. Forest plot showing odds ratios (ORs) and 95% CIs for A) cardiovascular disease, B) heart disease, and C) stroke associated with age, sex, marital status, place of residence, body mass index, smoking status, drinking status, physical activity; hypertension, history of chronic kidney disease, diabetes mellitus, and dyslipidemia. ORs, odds ratios; CVD, cardiovascular disease; SES, socioeconomic status; BMI, body mass index.

In the cross-sectional study, the prevalence of CVD was 16% (188/16,560) in the total population, with 23.8% (2,371/16,560) in high SES, 15.2% (827/16,560) in middle SES, and 8.2% (188/16,560) in low SES individuals (P for trend <0.001, S2 Table). After fully adjusting for sociodemographic and health-related factors, middle SES and low SES did not show statistically significant differences in association with CVD and its components (S2 Table, Fig 2). However, in the marginal structural model, middle SES [OR (95% CI): 1.77 (1.21–2.58)] was significantly associated with CVD (all P < 0.001, S5 Table), showing positive associations with both heart disease [1.66 (1.11–2.46)] and stroke [2.76 (1.18–6.44)] (all P < 0.05, S5 Table). Among the subset of 10,917 participants with metabolic biomarker measurements, further adjustments did not significantly change the cross-sectional relationship between SES and CVD (S5 Table).

### Longitudinal association between baseline SES status and incident cardiovascular disease at follow-up, 2015–2018

**Fig 3:**
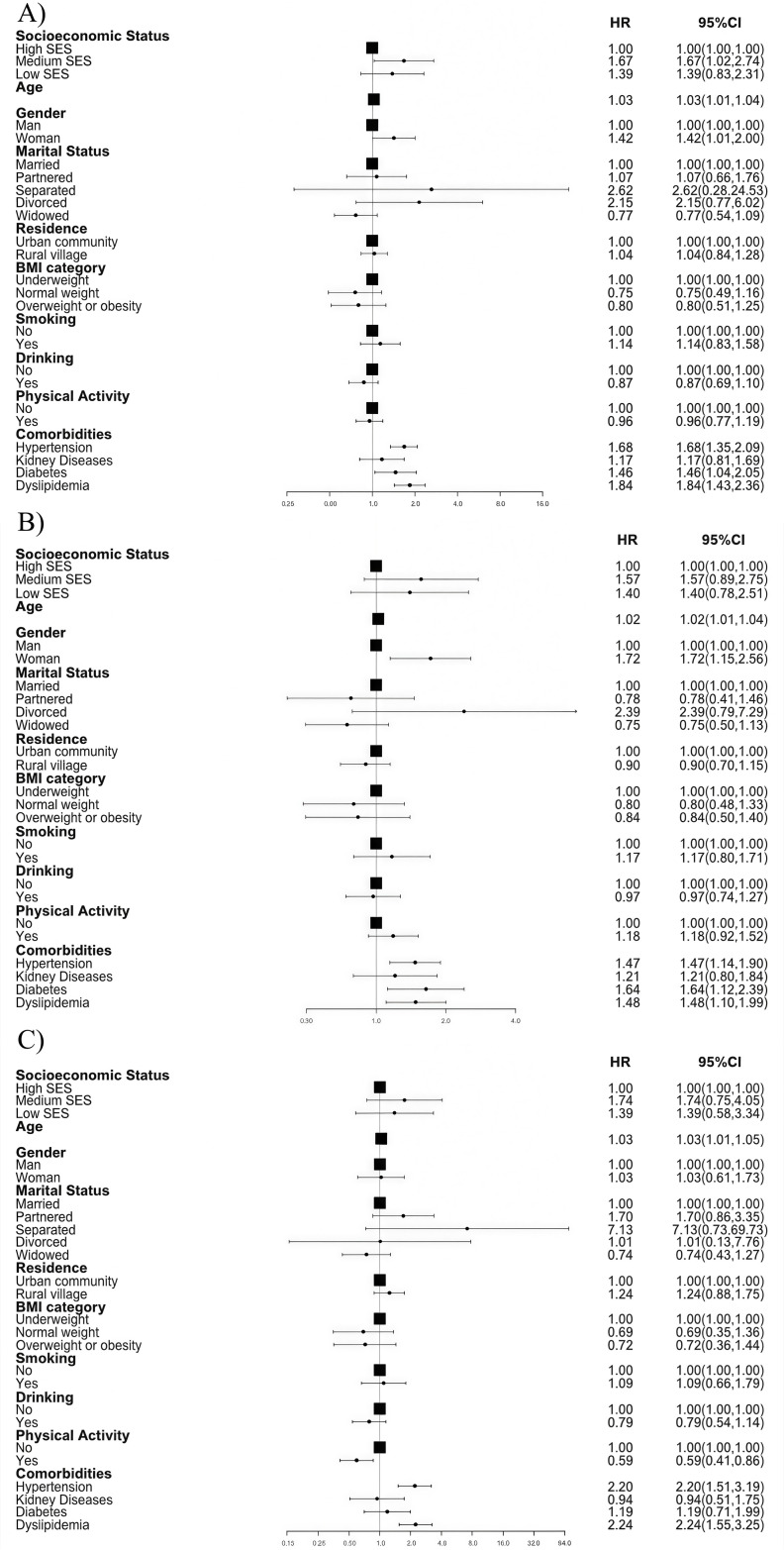
Longitudinal relationship between baseline socioeconomic status and cardiovascular disease incidence, 2015-2018. Forest plot showing odds ratios (ORs) and 95% CIs for A) cardiovascular disease, B) heart disease, and C) stroke associated with age, sex, marital status, place of residence, body mass index, smoking status, drinking status, physical activity; hypertension, history of chronic kidney disease, diabetes mellitus, and dyslipidemia. In Panel B, there is insufficient sample size in the “Separated” subcategory of Marital Status. Additionally, the population of “Never married” individuals is insufficient in Panel A–C. Therefore, this subcategory is not displayed in the figures. ORs, odds ratios; CVD, cardiovascular disease; SES, socioeconomic status; BMI, body mass index.

During the 3.0 years of follow-up, 1,404 cases (12.1%) of incident CVD events were identified, with an incidence rate of 40.39 per 1,000 person-years. Table 2 illustrates the relationship between baseline SES status and incident CVD. After adjusting for covariates in Models 3, individuals with middle SES were more likely to develop new-onset CVD compared to those with high SES [HR (95% CI): 1.67 (1.02–2.74), P < 0.05], (Table 2, Fig 3A). In the marginal structural model, the association between middle SES and CVD remained significant [HR (95% CI): 2.32 (1.33–4.06)], even after adjusting for metabolic biomarkers in a subpopulation of 7,910 subjects (S4 Table). Middle SES was associated with a 227% increased risk of CVD incidence [HR (95% CI): 2.27 (1.29–3.99), P < 0.05].

**Table 2:** Incidence of CVD according to socioeconomic status, 2015–2018.

Outcome	Cases, No.	Incidence Rate, per 1000 Person-Years	HR (95% CI)			
			Model 1^a^	Model 2^b^	Model 3^c^	Model 3plus^d^
CVD						
High SES	100	2.88	1.00 (Reference)	1.00 (Reference)	1.00 (Reference)	1.00 (Reference)
Middle SES	909	26.15	1.06 (0.85,1.32)	1.44 (1.01,2.06)^*^	1.67 (1.02,2.74)^*^	1.62 (0.99,2.65)
Low SES	395	11.36	0.89 (0.70,1.13)	1.17 (0.81,1.70)	1.39 (0.83,2.31)	1.37 (0.82,2.29)
Heart disease						
High SES	61	1.75	1.00 (Reference)	1.00 (Reference)	1.00 (Reference)	1.00 (Reference)
Middle SES	616	17.72	1.20 (0.91,1.59)	1.35 (0.89,2.05)	1.57 (0.89,2.75)	1.52 (0.87,2.68)
Low SES	275	7.91	1.04 (0.78,1.39)	1.17 (0.76,1.80)	1.40 (0.79,2.51)	1.37 (0.76,2.47)
Stroke						
High SES	42	1.21	1.00 (Reference)	1.00 (Reference)	1.00 (Reference)	1.00 (Reference)
Middle SES	344	9.90	0.91 (0.65,1.27)	1.68 (0.90,3.14)	1.74 (0.75,4.05)	1.66 (0.71,3.87)
Low SES	144	4.14	0.76 (0.53,1.09)	1.29 (0.68,2.48)	1.39 (0.58,3.34)	1.39 (0.58,3.35)

Abbreviation: HR, hazard ratio; CVD, cardiovascular disease; SES, socioeconomic status.

^a^Model 1 was adjusted for age, sex, marital status, residence.

^b^Model 2 was adjusted for age, sex, marital status, residence, smoking status, drinking status and physical activity.

^c^Model 3 was adjusted as model 2 with further adjustment for history of hypertension, dyslipidemia, diabetes and chronic kidney disease.

^d^Model 3 plus was adjusted as model 3 with further adjustment for triglycerides, creatinine, HDL cholesterol, LDL cholesterol, total cholesterol.

* P < 0.05.

### Sensitivity analysis

Using low SES as the reference group, we compared the longitudinal association of middle SES and high SES with CVD. Neither middle SES nor high SES showed a statistically significant association with CVD. However, in the marginal structural model, middle SES was positively associated with CVD [HR (95% CI): 1.32 (1.04–1.67), P < 0.05] (S4 Table).

## Discussion

This longitudinal and cross-sectional study analyzed information from the China Health and Retirement Longitudinal Study (CHARLS) for the years 2015 and 2018. It explores the connection between SES and CVD in China’s general population. The analysis reveals that SES is linked to the risk of CVD incidence. After adjusting for confounding factors, people with middle SES were found to have a significantly higher risk of cardiovascular disease. These findings were confirmed by a sensitivity analysis, which also showed that middle SES was strongly associated with CVD risk.

SES is an essential but often disregarded risk factor for CVD. A Chinese cohort study used education, occupation, and household wealth as indicators of SES and found that people of low SES, compared with those of high SES, had higher risks of all-cause mortality, cardiovascular disease mortality, non-cardiovascular disease mortality, major cardiovascular disease events, and cardiovascular disease hospitalizations compared to those with higher SES [36]. A population-based cohort on Chinese population examined how short-term changes in socioeconomic status affect long-term cardiovascular health and the research shows that improvements in socioeconomic status led to better heart health [37]. A population-based cohort study discovered that adults with low socioeconomic status in the US and UK had a higher risk of cardiovascular disease and death from any cause [17]. These findings emphasize the significant influence of socioeconomic status on heart health, stressing the importance of targeted efforts to reduce SES-related differences in cardiovascular disease outcomes.

Previous studies have widely acknowledged that low or middle socioeconomic status is linked to a higher risk of many diseases. A workshop review highlights significant disparities in CVD mortality and morbidity among different SES groups in the U.S [38]. Multiple studies have explored the association between low SES and CVD, finding individuals with low SES often face challenges in accessing healthcare, are more likely to engage in unhealthy habits like smoking and poor diet, and are exposed to harmful environmental factors such as noise and air pollution, which all contribute to the increased CVD risk [39–42]. In a multicohort study, researchers pooled individual-level data from six prospective cohort studies across 17 countries, they found low SES was associated with continuing or initiating physical inactivity, and continuing smoking [43]. A CARRS study explored the association between socioeconomic status and cardiovascular risk in urban South Asia and their result revealed that though SES-CVD patterns are heterogeneous, SES can be a risk factor for cardiovascular diseases [42]. In our present study, the data indicates middle SES was significantly associated with increased CVD risk, while sensitivity analysis confirmed these findings. Besides, the middle SES group exhibited a higher risk of CVD compared to the low SES group. This could be attributed to several factors, such as lifestyle behaviors (e.g., dietary habits, physical activity), access to healthcare, or the presence of intermediate risk factors like metabolic diseases, which may not have been fully adjusted for in our analysis. Additionally, individuals in the middle SES group might face unique stressors, such as job insecurity or unstable living conditions, that could exacerbate health risks. The results suggest that CVD risk does not always follow a linear gradient with SES, and the middle SES group might experience a complex interplay of factors that warrants further investigation. Future studies should explore this relationship in more depth and consider additional variables, such as mental health or social support, to better understand the underlying mechanisms. We acknowledge that potential biases may exist in the study, particularly with respect to the self-reported nature of some of the data, such as socioeconomic status (SES) and health conditions. To minimize these biases, we used validated instruments for data collection, and where possible, we cross-checked participant-reported information with available clinical data. Therefore, our study demonstrates that middle SES is associated with higher CVD risk, emphasizing the need for targeted interventions to address SES-related health disparities.

Several studies have confirmed the strong association between lower socioeconomic status (SES) and higher cardiovascular disease (CVD) risk. In high-income countries, lower SES groups face significantly higher rates of CVD and related risk factors. While lifestyle factors such as diet and physical activity can partly mediate this relationship, they explain only a small portion of the disparities, suggesting that broader interventions addressing social determinants of health are essential. Additionally, cultural and contextual factors, as seen in rural Spain, may play a more significant role than education alone in shaping cardiovascular risk. This highlights the need for tailored, context-specific interventions. Given the rising CVD burden in low- and middle-income countries, more longitudinal studies are needed to better understand the impact of SES on CVD in these regions. Overall, reducing CVD disparities requires a multifaceted approach that includes both lifestyle changes and addressing underlying social factors [17,41,44–47].

Our findings show high cardiovascular risk in people of middle socioeconomic status instead of low socioeconomic status. Although the 3-year follow-up period may be insufficient to observe the long-term effects of SES changes, the high incidence of CVD events in the Chinese population allows for the detection of significant associations even in the medium-term follow-up [48]. In this regard, we propose a hypothesis that the improvement may be due to the effective policies implemented by the Chinese government to upgrade primary healthcare services. China has established a primary healthcare network across urban and rural areas, ensuring grassroots access to basic medical services close to home [49]. Implementation of the New Rural Cooperative Medical System (NCMS), which provides basic medical protection for rural residents, reduces the burden of medical care on the public through medical insurance and other means, and alleviates the phenomenon of poverty arising from illness and returning to poverty [50,51]. The National Basic Public Health Service Program (NBPHSP) was launched to provide urban and rural residents with a range of basic public health services, such as vaccination, chronic disease management, and maternal and child health care, free of charge [52]. However, the growth of the middle socioeconomic status group is crucial for social stability and economic development, as it plays a key role in the consumer market and contributes significantly to social progress, especially in developing countries [53,54]. Therefore, extending the follow-up period in future studies is essential, as it will enable the capture of more outcome events and facilitate a deeper investigation. We also hypothesize that individuals in the middle SES group may face unique stressors or health risks, such as limited access to healthcare, financial insecurity, or a lack of social support, which could contribute to an elevated CVD risk. Additionally, the middle SES group may experience lifestyle behaviors such as diet, physical inactivity and stress that are different from those in both higher and lower SES groups, which could further explain this unexpected association.

Recent literature highlights the critical role of social determinants of health (SDOH) in shaping cardiovascular disease (CVD) disparities. These factors, including economic stability, education, healthcare access, and neighborhood environment, contribute significantly to CVD risk and outcomes. Several studies underscore the disproportionate burden of CVD on socially disadvantaged populations, particularly racial and ethnic minorities, due to structural inequities and systemic barriers. Interventions targeting SDOH such as education, housing, income supplements, and community development have shown promise in reducing health disparities and improving cardiovascular outcomes. However, existing frameworks largely overlook the cumulative impact of multiple SDOH domains on CVD health, particularly among marginalized groups. Moreover, there is a need for more comprehensive studies and cost-effectiveness analyses to better inform policy decisions and resource allocation. Addressing these disparities requires a multi-faceted approach that includes both upstream social interventions and targeted health policies. Interdisciplinary efforts are essential to deepen the understanding of the biological mechanisms linking SDOH to CVD, including stress, inflammation, and immune function. These findings support the importance of integrating SDOH into CVD prevention and care strategies to promote health equity [55–59].

Cardiovascular diseases have become a major disease burden in China, and given the dual pressures of an aging population and a steadily increasing prevalence of metabolic risk factors, the burden caused by cardiovascular diseases will continue to increase, which poses new requirements for the prevention and treatment of cardiovascular diseases and the allocation of medical resources in China [60]. Different policies for CVD prevention for different populations are necessary to more effectively address socioeconomic disparities in CVD and improve the health of the population. Our findings support the need for policy makers to make trade-offs between middle and lower socioeconomic status groups when formulating policies to ensure that they reach all segments of society. This could facilitate more precise targeting of resources for public health services, but more long-term prospective studies are needed to further elucidate and solidify the relationship between changes in socioeconomic status and cardiovascular disease in China.

## Conclusion

In the general population of China, middle socioeconomic status (SES) is positively associated with cardiovascular disease (CVD) and is more likely to be linked to new-onset CVD. These findings suggest a need for balanced, targeted interventions across different SES groups to effectively reduce CVD risk, with particular attention to the often overlooked middle SES group. Future policies should consider trade-offs that address the unique vulnerabilities of each SES group while optimizing overall health outcomes. Additionally, further long-term prospective studies are essential to clarify the dynamic relationship between changes in SES and CVD in China, while healthcare providers should also consider tailoring interventions based on the unique challenges faced by different SES groups, focusing on both prevention and management strategies for CVD. Future efforts should integrate broader social support systems and community resources to improve cardiovascular health outcomes across all SES groups.

### Strengths and limitations

Our study has several strengths. First, our study not only focused on the cross-sectional association between socioeconomic status and cardiovascular disease, but also explored the longitudinal association between socioeconomic status and the risk of new-onset cardiovascular disease, but also used nationally representative data, which led to more reliable conclusions and provided more robust evidence to explore the relationship between socioeconomic status and cardiovascular disease. Second, multiple socioeconomic status indicators were considered, reflecting the complexity of socioeconomic status more comprehensively. Finally, potential confounders were adjusted to make the findings more accurate.

However, there are some limitations to this study. Primarily, the study was limited to the Chinese population and may not be directly generalizable to other regions or ethnicities. In addition, the follow-up period was relatively short which limits the ability to observe long-term trends or changes in CVD risk. Finally, the findings may not be generalizable to populations outside of China as the result of the unique Chinese healthcare system and socioeconomic conditions. It is also important to acknowledge the limitations of the self-reported data and the simplified socioeconomic status (SES) measures used in this study, which may limit the precision of our SES assessment, particularly in a country as socioeconomically diverse as China. Consequently, the generalizability of our results to other populations, particularly those with more varied socioeconomic backgrounds, may be limited. Future studies should consider employing more detailed SES measures and objective data collection methods to better assess the nuanced relationship between SES and cardiovascular disease (CVD) outcomes and to improve the robustness and generalizability of findings.

**Key Points**

**Question**

Is socioeconomic status associated with the risk of cardiovascular events and new cardiovascular events in the general Chinese population?

**Findings**

Our study found that middle socioeconomic status was positively associated with the risk of cardiovascular events and new cardiovascular events.

**Meaning**

Our findings support the need for trade-offs between socioeconomic status groups to benefit different populations, especially considering the middle socioeconomic status group, which is an easily overlooked group.

## Supporting information

S1 Table Baseline characteristics of participants without CVD by socioeconomic status in the longitudinal analysis. Data are shown as means ± standard deviation or numbers (percentages). Categorical variables were analyzed using chi-square tests, and continuous variables were analyzed using ANOVA. Abbreviation: BMI, body mass index; LDL, low-density lipoprotein; HDL, high-density lipoprotein.
(DOCX)

S2 Table Cross-sectional associations between socioeconomic status, CVD and its components among all participants. Abbreviation: OR, Odds ratio; CVD, cardiovascular disease;SES,socioeconomic status. a Model 1 was adjusted for age, sex, marital status, residence. b Model 2 was adjusted for age, sex, marital status, residence, smoking status, drinking status and physical activity. c Model 3 was adjusted as model 2 with further adjustment for history of hypertension, dyslipidemia, diabetes and chronic kidney disease. d Model 3 plus was adjusted as model 3 with further adjustment for triglycerides, creatinine, HDL cholesterol, LDL cholesterol, total cholesterol. Categorical variables were analyzed using chi-square tests, and continuous variables were analyzed using ANOVA. *P < 0.05. **P < 0.001.
(DOCX)

S3 Table Incidence of CVD according to socioeconomic status, 2015–2018 (Low SES as a control group). Abbreviation: HR, hazard ratio; CVD, cardiovascular disease;SES,socioeconomic status. a Model 1 was adjusted for age, sex, marital status, residence. b Model 2 was adjusted for age, sex, marital status, residence, smoking status, drinking status and physical activity. c Model 3 was adjusted as model 2 with further adjustment for history of hypertension, dyslipidemia, diabetes and chronic kidney disease. Categorical variables were analyzed using chi-square tests, and continuous variables were analyzed using ANOVA. *P < 0.05.
(DOCX)

S4 Table Incidence of CVD according to socioeconomic status, 2015–2018(MSM). Abbreviation: HR, hazard ratio; CVD, cardiovascular disease;SES,socioeconomic status;MSM,Marginal structural models. a Model 1 was adjusted for age, sex, marital status, residence. B Model 2 was adjusted for age, sex, marital status, residence, smoking status, drinking status and physical activity. c Model 3 was adjusted as model 2 with further adjustment for history of hypertension, dyslipidemia, diabetes and chronic kidney disease. d Model 3 plus was adjusted as model 3 with further adjustment for triglycerides, creatinine, HDL cholesterol, LDL cholesterol, total cholesterol. *P < 0.05. **P < 0.01. ***P < 0.001.
(DOCX)

S5 Table Cross-sectional associations between socioeconomic status and cardiovascular disease (MSM). Abbreviation: OR, Odds ratio; CVD, cardiovascular disease;SES,socioeconomic status;MSM,Marginal structural models. a Model 1 was adjusted for age, sex, marital status, residence. b Model 2 was adjusted for age, sex, marital status, residence, smoking status, drinking status and physical activity. c Model 3 was adjusted as model 2 with further adjustment for history of hypertension, dyslipidemia, diabetes and chronic kidney disease. *P < 0.05. **P < 0.01. ***P < 0.001.
(DOCX)

S6 Table Latent Class Ananlysis (using poLCA package). N.Class = 4. * LMR: ad-hoc adjusted likelihood ratio test (LRT) described in Formula 15 of Lo, Mendell, & Rubin (2001).
(DOCX)

## Abbreviations

CVD: cardiovascular disease
SES: socioeconomic status
BMI: body mass index
HR: hazard ratio
OR: odds ratio
SD: standard deviation
MSM: marginal structural models.
