# Supplementary material for: Longitudinal associations between socioeconomic status and cardiovascular disease in a Chinese population: Evidence from CHARLS
Source: PLoS One. 2025 Aug 22;20(8):e0328924. doi: 10.1371/journal.pone.0328924 (PMC12373183; doi:10.1371/journal.pone.0328924)
Supplement: S1 Table — Data are shown as means ± standard deviation or numbers (percentages). Categorical variables were analyzed using chi-square tests, and continuous variables were analyzed using ANOVA. Abbreviation: BMI, body mass index; LDL, low-density lipoprotein; HDL, high-density lipoprotein. (DOCX) [file pone.0328924.s001.docx]

S1 Table. Baseline characteristics of participants without CVD by socioeconomic status in the longitudinal analysis

| Characteristics | Total（n=11587） | socioeconomic status | | | P-value |
| --- | --- | --- | --- | --- | --- |
|  |  | High  （n=876） | Medium  （n=6654） | Low  （n=4057） |  |
| Age(y) | 60.2±9.3 | 58.1±8.4 | 62.6±9.6 | 56.8±7.6 | <0.001 |
| Gender |  |  |  |  | <0.001 |
| Man | 5643(48.7%) | 437(49.9%) | 2677(40.2%) | 2529 (62.3%) |  |
| Woman | 5944(51.3%) | 439(50.1%) | 3977(59.8%) | 1528(37.7%) |  |
| Educational level |  |  |  |  | <0.001 |
| Less than high school | 10333 (89.2%) | 762(87.0%) | 6538 (98.3%) | 3033 (74.8%) |  |
| High school or  equivalent | 1085(9.4%) | 92(10.5%) | 0(0.0%) | 993 (24.5%) |  |
| College or above | 169(1.5%) | 22(2.5%) | 116(1.7%) | 31 (0.8%) |  |
| Health insurance |  |  |  |  | <0.001 |
| No | 974(8.6%) | 606(70.9%) | 349(5.3%) | 19 (0.5%) |  |
| Public | 10045(89.1%) | 0 (0.0%) | 6173(94.6%) | 3872(99.4%) |  |
| Private | 258(2.3%) | 249(29.1%) | 3 (0.0%) | 6(0.2%) |  |
| Incomes category |  |  |  |  | <0.001 |
| Low | 954(39.7%) | 53(22.3%) | 15(12.8%) | 886(43.3%) |  |
| Medium | 799 (33.3%) | 86(36.1%) | 0(0.0%) | 713(34.8%) |  |
| High | 649(27.0%) | 99(41.6%) | 102(87.2%) | 448(21.9%) |  |
| Occupation |  |  |  |  | <0.001 |
| Unemployment or retirement | 3485(30.2%) | 102(11.7%) | 2955(44.4%) | 428(10.7%) |  |
| Agriculture | 4526(39.3%) | 414(47.4%) | 3648(54.9%) | 464(11.6%) |  |
| Nonagriculture | 3511(30.5%) | 357(40.9%) | 46 (0.7%) | 3108(77.7%) |  |
| Marital status |  |  |  |  | <0.001 |
| Married | 9475(81.8%) | 733 (83.7%) | 5326(80.0%) | 3416(84.2% |  |
| Partnered | 697(6.0%) | 48(5.5%) | 309(4.6%) | 340(8.4%) |  |
| Separated | 22(0.2%) | 4(0.5% | 11(0.2%) | 7(0.2%) |  |
| Divorced | 79 (0.7%) | 12(1.4%) | 36(0.5%) | 31(0.8%) |  |
| Widowed | 1241(10.7%) | 70(8.0%) | 928(13.9%) | 243(6.0%) |  |
| Never married | 73(0.6%) | 9(1.0%) | 44 (0.7%) | 20(0.5%) |  |
| Residence |  |  |  |  | <0.001 |
| Urban community | 4113(35.5%) | 339(38.7%) | 2053(30.9%) | 1721(42.4%) |  |
| Rural village | 7474(64.5%) | 537(61.3%) | 4601(69.1%) | 2336(57.6%) |  |
| BMI category |  |  |  |  | <0.001 |
| Underweight | 542(5.7%) | 37 (5.2%) | 378(6.7%) | 127(4.1%) |  |
| Normal weight | 4797(50.6% | 376(53.3%) | 2893(51.2%) | 1528(48.9%) |  |
| Overweight or obesity | 4135(43.6%) | 293(41.5%) | 2374(42.1%) | 1468(47.0%) |  |
| Smoking |  |  |  |  | <0.001 |
| No | 6465(55.8%) | 479(54.7%) | 4075(61.2% | 1911(47.1% |  |
| Yes | 5119(44.2%) | 396(45.3%) | 2579(38.8%) | 2144(52.9%) |  |
| Drinking |  |  |  |  | <0.001 |
| No | 6171 (53.3%) | 413(47.2%) | 3939 (59.3%`) | 1819(44.9%) |  |
| Yes | 5398(46.7%) | 462(52.8%) | 2705(40.7%) | 2231 (55.1%) |  |
| Physical Activity or Exercise |  |  |  |  | <0.001 |
| No | 3455(61.8%) | 237(57.7%) | 2086(64.8%) | 1132(57.6%) |  |
| Yes | 2137(38.2%) | 174(42.3%) | 1131(35.2%) | 832(42.4%) |  |
| Triglycerides (mg/dl) | 140.3±89.0 | 139.6±90.7 | 138.5±86.8 | 143.7±92.6 | 0.062 |
| Creatinine (mg/dl) | 0.8±0.3 | 0.8±0.2 | 0.8±0.2 | 0.8±0.3 | <0.001 |
| HDL cholesterol (mg/dl) | 51.7±11.6 | 52.7±12.0 | 51.9±11.5 | 51.1±11.6 | 0.001 |
| LDL cholesterol (mg/dl) | 102.8±29.0 | 102.3±28.2 | 103.8±29.1 | 101.0±28.9 | <0.001 |
| Total cholesterol (mg/dl) | 184.5±36.6 | 184.5±34.2 | 185.6±37.2 | 182.3±35.9 | 0.001 |
| Glucose (mg/dl) | 103.2±34.2 | 102.4±33.7 | 103.2±33.2 | 103.4±36.1 | 0.821 |
| Hypertension |  |  |  |  | <0.001 |
| No | 4511(46.5%) | 370(51.5%) | 2581(44.6%) | 1560(48.8%) |  |
| Yes | 5188(53.5%) | 348(48.5%) | 3200(55.4%) | 1640(51.2%) |  |
| Dyslipidemia |  |  |  |  | 0.240 |
| No | 9621 (85.8%) | 726(86.2%) | 5535(86.2%) | 3360(85.1%) |  |
| Yes | 1590(14.2%) | 116(13.8%) | 884(13.8%) | 590 (14.9%) |  |
| Heart diseases |  |  |  |  | <0.001 |
| No | 10635(91.8%) | 815 (93.0%) | 6038 (90.7%) | 3782(93.2%) |  |
| Yes | 952(8.2%) | 61(7.0%) | 616(9.3%) | 275(6.8%) |  |
| Stroke |  |  |  |  | <0.001 |
| No | 11057 (95.4%) | 834(95.2%) | 6310(94.8%) | 3913 (96.5%) |  |
| Yes | 530(4.6%) | 42(4.8%) | 344(5.2%) | 144(3.5%) |  |
| Cardiovascular disease |  |  |  |  | <0.001 |
| No | 10183(87.9%) | 776(88.6%) | 5745(86.3%) | 3662(90.3%) |  |
| Yes | 1404(12.1%) | 100(11.4%) | 909 (13.7%) | 395(9.7%) |  |
| Kidney Diseases |  |  |  |  | 0.937 |
| No | 10657 (92.5%) | 805 (92.8%) | 6111 (92.5%) | 3741 (92.5%) |  |
| Yes | 859 (7.5%) | 62 (7.2%) | 495 (7.5%) | 302 (7.5%) |  |

Data are shown as means ± standard deviation or numbers (percentages). Categorical variables were analyzed using chi-square tests, and continuous variables were analyzed using ANOVA.

Abbreviation: BMI, body mass index; LDL, low-density lipoprotein; HDL, high-density lipoprotein.
