# Supplementary material for: Longitudinal associations between socioeconomic status and cardiovascular disease in a Chinese population: Evidence from CHARLS
Source: PLoS One. 2025 Aug 22;20(8):e0328924. doi: 10.1371/journal.pone.0328924 (PMC12373183; doi:10.1371/journal.pone.0328924)
Supplement: S2 Table — Abbreviation: OR, Odds ratio; CVD, cardiovascular disease;SES,socioeconomic status. a Model 1 was adjusted for age, sex, marital status, residence. b Model 2 was adjusted for age, sex, marital status, residence, smoking status, drinking status and physical activity. c Model 3 was adjusted as model 2 with further adjustment for history of hypertension, dyslipidemia, diabetes and chronic kidney disease. d Model 3 plus was adjusted as model 3 with further adjustment for triglycerides, creatinine, HDL cholesterol, LDL cholesterol, total cholesterol. Categorical variables were analyzed using chi-square tests, and continuous variables were analyzed using ANOVA. *P < 0.05. **P < 0.001. (DOCX) [file pone.0328924.s002.docx]

S2 Table. Cross-sectional associations between socioeconomic status , CVD and its components among all participants.

| Outcome | Cases, n (%) | OR (95% CI) | | |  |
| --- | --- | --- | --- | --- | --- |
|  |  | Model 1^a^ | Model 2^b^ | Model 3^c^ | Model 3plus^d^ |
| CVD |  |  |  |  |  |
| High SES | 188(16.0%) | 1.00 (Reference) | 1.00 (Reference) | 1.00 (Reference) | 1.00 (Reference) |
| Medium SES | 2371(23.8%) | 1.39  (1.18,1.64)** | 1.32  (1.03,1.69)* | 1.23  (0.88,1.72) | 1.21  (0.86,1.69) |
| Low SES | 827(15.2%) | 1.00  (0.84,1.19) | 0.97  (0.75,1.26) | 0.94  (0.66,1.34) | 0.93  (0.65,1.32) |
| Heart disease |  |  |  |  |  |
| High SES | 161(13.7%) | 1.00 (Reference) | 1.00 (Reference) | 1.00 (Reference) | 1.00 (Reference) |
| Medium SES | 2057(20.7%) | 1.41  (1.18,1.68)** | 1.30  (1.00,1.68) | 1.18  (0.83,1.67) | 1.15  (0.81,1.64) |
| Low SES | 743(13.7%) | 1.07  (0.89,1.29) | 1.04  (0.80,1.37) | 0.98  (0.68,1.41) | 0.97  (0.67,1.40) |
| Stroke |  |  |  |  |  |
| High SES | 38(3.2%) | 1.00 (Reference) | 1.00 (Reference) | 1.00 (Reference) | 1.00 (Reference) |
| Medium SES | 475(4.8%) | 1.25  (0.89,1.76) | 1.20  (0.72,2.01) | 1.42  (0.65,3.12) | 1.40  (0.64,3.08) |
| Low SES | 129(2.4%) | 0.72  (0.50,1.05) | 0.54  (0.31,0.95)* | 0.76  (0.33,1.78) | 0.70  (0.30,1.64) |

Abbreviation: OR, Odds ratio; CVD, cardiovascular disease;SES,socioeconomic status.

a Model 1 was adjusted for age, sex, marital status, residence.

b Model 2 was adjusted for age, sex, marital status, residence, smoking status, drinking status and physical activity.

c Model 3 was adjusted as model 2 with further adjustment for history of hypertension, dyslipidemia, diabetes and chronic kidney disease.

d Model 3 plus was adjusted as model 3 with further adjustment for triglycerides, creatinine, HDL cholesterol, LDL cholesterol, total cholesterol.

Categorical variables were analyzed using chi-square tests, and continuous variables were analyzed using ANOVA.

*P < 0.05.

**P < 0.001.
