# Supplementary material for: Longitudinal associations between socioeconomic status and cardiovascular disease in a Chinese population: Evidence from CHARLS
Source: PLoS One. 2025 Aug 22;20(8):e0328924. doi: 10.1371/journal.pone.0328924 (PMC12373183; doi:10.1371/journal.pone.0328924)
Supplement: S3 Table — Abbreviation: HR, hazard ratio; CVD, cardiovascular disease;SES,socioeconomic status. a Model 1 was adjusted for age, sex, marital status, residence. b Model 2 was adjusted for age, sex, marital status, residence, smoking status, drinking status and physical activity. c Model 3 was adjusted as model 2 with further adjustment for history of hypertension, dyslipidemia, diabetes and chronic kidney disease. Categorical variables were analyzed using chi-square tests, and continuous variables were analyzed using ANOVA. *P < 0.05. (DOCX) [file pone.0328924.s003.docx]

S3 Table. Incidence of CVD according to socioeconomic status, 2015–2018（Low SES as a control group）.

| Outcome | Cases, n (%) | Incidence Rate, per 1000 Person-Years | HR (95% CI) | | |
| --- | --- | --- | --- | --- | --- |
|  |  |  | Model 1^a^ | Model 2^b^ | Model 3^c^ |
| CVD |  |  |  |  |  |
| Low SES | 395 | 11.36 | 1.00 (Reference) | 1.00 (Reference) | 1.00 (Reference) |
| Medium SES | 909 | 26.15 | 1.19  (1.04,1.36)* | 1.23  (1.02,1.49)* | 1.21  (0.95,1.53) |
| High SES | 100 | 2.88 | 1.12  (0.89,1.42) | 0.85  (0.59,1.24) | 0.72  (0.43,1.20) |
| Heart disease |  |  |  |  |  |
| Low SES | 275 | 7.91 | 1.00 (Reference) | 1.00 (Reference) | 1.00 (Reference) |
| Medium SES | 616 | 17.72 | 1.15  (0.98,1.35) | 1.15  (0.92,1.44) | 1.12  (0.85,1.48) |
| High SES | 61 | 1.75 | 0.96  (0.72,1.28) | 0.85  (0.56,1.31) | 0.72  (0.40,1.28) |
| Stroke |  |  |  |  |  |
| Low SES | 144 | 4.14 | 1.00 (Reference) | 1.00 (Reference) | 1.00 (Reference) |
| Medium SES | 344 | 9.90 | 1.19  (0.96,1.48) | 1.30  (0.95,1.77) | 1.25  (0.85,1.85) |
| High SES | 42 | 1.21 | 1.31  (0.92,1.87) | 0.77  (0.40,1.48) | 0.72  (0.30,1.73) |

Abbreviation: HR, hazard ratio; CVD, cardiovascular disease;SES,socioeconomic status.

a Model 1 was adjusted for age, sex, marital status, residence.

b Model 2 was adjusted for age, sex, marital status, residence, smoking status, drinking status and physical activity.

c Model 3 was adjusted as model 2 with further adjustment for history of hypertension, dyslipidemia, diabetes and chronic kidney disease.

Categorical variables were analyzed using chi-square tests, and continuous variables were analyzed using ANOVA.

*P < 0.05.
