# Supplementary material for: Longitudinal associations between socioeconomic status and cardiovascular disease in a Chinese population: Evidence from CHARLS
Source: PLoS One. 2025 Aug 22;20(8):e0328924. doi: 10.1371/journal.pone.0328924 (PMC12373183; doi:10.1371/journal.pone.0328924)
Supplement: S4 Table — Abbreviation: HR, hazard ratio; CVD, cardiovascular disease;SES,socioeconomic status;MSM,Marginal structural models. a Model 1 was adjusted for age, sex, marital status, residence. B Model 2 was adjusted for age, sex, marital status, residence, smoking status, drinking status and physical activity. c Model 3 was adjusted as model 2 with further adjustment for history of hypertension, dyslipidemia, diabetes and chronic kidney disease. d Model 3 plus was adjusted as model 3 with further adjustment for triglycerides, creatinine, HDL cholesterol, LDL cholesterol, total cholesterol. *P < 0.05. **P < 0.01. ***P < 0.001. (DOCX) [file pone.0328924.s004.docx]

S4 Table. Incidence of CVD according to socioeconomic status, 2015–2018(MSM).

| Outcome | Cases, n (%) | Incidence Rate, per 1000 Person-Years | HR (95% CI) | | | |
| --- | --- | --- | --- | --- | --- | --- |
|  |  |  | Model 1^a^ | Model 2^b^ | Model 3^c^ | Model 3 plus^d^ |
| CVD | 1404 | 40.39 |  |  |  |  |
| High SES | 100 | 2.88 | 1.00 (Reference) | 1.00 (Reference) | 1.00 (Reference) | 1.00 (Reference) |
| Medium SES | 909 | 26.15 | 1.37  (1.06,1.77)* | 1.81  (1.23,2.68)** | 2.32  (1.33,4.06)** | 2.27  (1.29,3.99)** |
| Low SES | 395 | 11.36 | 1.09  (0.83,1.44) | 1.36  (0.90,2.05) | 1.76  (0.98,3.16) | 1.75  (0.97,3.14） |
| Heart disease | 952 | 27.39 |  |  |  |  |
| High SES | 61 | 1.75 | 1.00 (Reference) | 1.00 (Reference) | 1.00 (Reference) | 1.00 (Reference) |
| Medium SES | 616 | 17.72 | 1.54  (1.11,2.14)** | 1.68  (1.07,2.63)* | 2.11  (1.10,4.04) | 2.08  (1.08,4.01)* |
| Low SES | 275 | 7.91 | 1.27  (0.90,1.80) | 1.37  (0.86,2.19) | 1.75  (0.89,3.42) | 1.72  (0.87,3.40) |
| Stroke | 530 | 15.25 |  |  |  |  |
| High SES | 42 | 1.21 | 1.00 (Reference) | 1.00 (Reference) | 1.00 (Reference) | 1.00 (Reference) |
| Medium SES | 344 | 9.90 | 1.18  (0.81,1.72) | 2.20  (1.10,4.39)* | 2.72  (1.04,7.15)* | 2.67  (1.02,6.98)* |
| Low SES | 144 | 4.14 | 0.93  (0.62,1.39) | 1.52  (0.74,3.15) | 1.94  (0.71,5.34) | 1.97  (0.72,5.39) |

Abbreviation: HR, hazard ratio; CVD, cardiovascular disease;SES,socioeconomic status;MSM,Marginal structural models.

a Model 1 was adjusted for age, sex, marital status, residence.

B Model 2 was adjusted for age, sex, marital status, residence, smoking status, drinking status and physical activity.

c Model 3 was adjusted as model 2 with further adjustment for history of hypertension, dyslipidemia, diabetes and chronic kidney disease.

d Model 3 plus was adjusted as model 3 with further adjustment for triglycerides, creatinine, HDL cholesterol, LDL cholesterol, total cholesterol.

*P < 0.05

**P < 0.01.

***P < 0.001
