# Supplementary material for: Longitudinal associations between socioeconomic status and cardiovascular disease in a Chinese population: Evidence from CHARLS
Source: PLoS One. 2025 Aug 22;20(8):e0328924. doi: 10.1371/journal.pone.0328924 (PMC12373183; doi:10.1371/journal.pone.0328924)
Supplement: S5 Table — Abbreviation: OR, Odds ratio; CVD, cardiovascular disease;SES,socioeconomic status;MSM,Marginal structural models. a Model 1 was adjusted for age, sex, marital status, residence. b Model 2 was adjusted for age, sex, marital status, residence, smoking status, drinking status and physical activity. c Model 3 was adjusted as model 2 with further adjustment for history of hypertension, dyslipidemia, diabetes and chronic kidney disease. *P < 0.05. **P < 0.01. ***P < 0.001. (DOCX) [file pone.0328924.s005.docx]

S5 Table. Cross-sectional associations between socioeconomic status and cardiovascular disease（MSM）

| Outcome | Cases, n (%) | OR (95% CI) | | | |
| --- | --- | --- | --- | --- | --- |
|  |  | Model 1^a^ | Model 2^b^ | Model 3^c^ | Model 3plus^d^ |
| CVD |  |  |  |  |  |
| High SES | 188(16.0%) | 1.00 (Reference) | 1.00 (Reference) | 1.00 (Reference) | 1.00 (Reference) |
| Medium SES | 2371(23.8%) | 1.84  (1.52,2.23)** | 1.81  (1.36,2.41)** | 1.77  (1.21,2.58)** | 2.27  (1.29,3.99)* |
| Low SES | 827(15.2%) | 1.23  (1.00,1.50)* | 1.23  (0.91,1.66) | 1.24  (0.83,1.84) | 1.75  (0.97,3.14) |
| Heart disease |  |  |  |  |  |
| High SES | 161(13.7%) | 1.00 (Reference) | 1.00 (Reference) | 1.00 (Reference) | 1.00 (Reference) |
| Medium SES | 2057(20.7%) | 1.89  (1.54,2.32) | 1.84  (1.35,2.49)*** | 1.66  (1.11,2.46)* | 2.08  (1.08,4.01)* |
| Low SES | 743(13.7%) | 1.34  (1.08,1.66)** | 1.36  (0.99,1.87) | 1.25  (0.82,1.90) | 1.72  (0.87,3.40) |
| Stroke |  |  |  |  |  |
| High SES | 38(3.2%) | 1.00 (Reference) | 1.00 (Reference) | 1.00 (Reference) | 1.00 (Reference) |
| Medium SES | 475(4.8%) | 1.55  (1.05,2.29)* | 1.43  (0.79,2.57) | 2.76  (1.18,6.44)* | 2.67  (1.02,6.98)* |
| Low SES | 129(2.4%) | 0.83  (0.54,1.28) | 0.60  (0.31,1.15) | 1.35  (0.54,3.38) | 1.97  (0.72,5.39) |

Abbreviation: OR, Odds ratio; CVD, cardiovascular disease;SES,socioeconomic status;MSM,Marginal structural models.

a Model 1 was adjusted for age, sex, marital status, residence.

b Model 2 was adjusted for age, sex, marital status, residence, smoking status, drinking status and physical activity.

c Model 3 was adjusted as model 2 with further adjustment for history of hypertension, dyslipidemia, diabetes and chronic kidney disease.

*P < 0.05.

**P < 0.01.

***P < 0.001
