# Supplementary material for: Longitudinal associations between socioeconomic status and cardiovascular disease in a Chinese population: Evidence from CHARLS
Source: PLoS One. 2025 Aug 22;20(8):e0328924. doi: 10.1371/journal.pone.0328924 (PMC12373183; doi:10.1371/journal.pone.0328924)
Supplement: S6 Table — N.Class = 4. * LMR: ad-hoc adjusted likelihood ratio test (LRT) described in Formula 15 of Lo, Mendell, & Rubin (2001). (DOCX) [file pone.0328924.s006.docx]

S6 Table. Latent Class Ananlysis (using poLCA package)

| N.Class | Entropy.R2 | BIC | AIC | Max. Log-likelihood | #Parameters | Residual df. | * Log-likelihood ratio test |
| --- | --- | --- | --- | --- | --- | --- | --- |
| 1 |  | 475405.5 | 475329.3 | -237656.660 | 8 | 72 |  |
| 2 | 0.110 | 475964.2 | 475802.2 | -237884.120 | 17 | 63 | LR: -454.9 LMR.LR: -438.7 df: 9 LMR.P: =1.0000 |
| 3 | 0.313 | 453602.1 | 453354.4 | -226651.200 | 26 | 54 | LR: 22465.8 LMR.LR: 21667.1 df: 9 LMR.P: <0.0001 |
| 4 | 0.348 | 452989.9 | 452656.5 | -226293.230 | 35 | 45 | LR: 715.9 LMR.LR: 690.5 df: 9 LMR.P: <0.0001 |
| 5 | 0.113 | 469765.1 | 469345.9 | -234628.960 | 44 | 36 | LR: -16671.5 LMR.LR: -16078.7 df: 9 LMR.P: =1.0000 |

N.Class = 4 
* LMR: ad-hoc adjusted likelihood ratio test (LRT) described in Formula 15 of Lo, Mendell, & Rubin (2001)
